# Novel Anticancer Platinum(IV) Complexes with Adamantylamine: Their Efficiency and Innovative Chemotherapy Strategies Modifying Lipid Metabolism

**DOI:** 10.1155/2008/417897

**Published:** 2008-04-08

**Authors:** Alois Kozubík, Alena Vaculová, Karel Souček, Jan Vondráček, Jaroslav Turánek, Jiřina Hofmanová

**Affiliations:** ^1^Department of Cytokinetics, Institute of Biophysics, Královopolská 135, 612 65 Brno, Czech Republic; ^2^Department of Vaccinology and Immunotherapy, Veterinary Research Institute, Hudcova 70, 621 00 Brno, Czech Republic

## Abstract

The impressive impact of cisplatin on cancer on one side and severe side effects, as well as the development of drug resistance during treatment on the other side, were the factors motivating scientists to design and synthesize new more potent analogues lacking disadvantages of cisplatin. Platinum(IV) complexes represent one of the perspective groups of platinum-based drugs. In this review, we summarize recent findings on both *in vitro* and *in vivo* effects of platinum(IV) complexes with adamantylamine. Based on a literary overview of the mechanisms of activity of platinum-based cytostatics, we discuss opportunities for modulating the effects of novel platinum complexes through interactions with apoptotic signaling pathways and with cellular lipids, including modulations of the mitochondrial cell death pathway, oxidative stress, signaling of death ligands, lipid metabolism/signaling, or intercellular communication. These approaches might significantly enhance the efficacy of both novel and established platinum-based cytostatics.

## 1. INTRODUCTION

**Figure 1:**
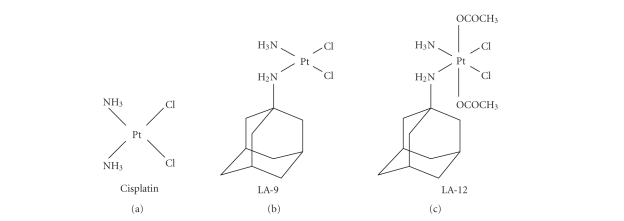
Chemical structures of cisplatin [cis-diamminedichloroplatinum(II)], LA-9 [(SP-4-3)-(1-adamantylamine)amminedichloroplatinum(II)], and LA-12 [(OC-6-43)-bis(acetato)(1-adamantylamine) amminedichloroplatinum(IV)].

Platinum-based drugs are widely used anticancer agents with a broad range of antitumor activities. Cisplatin [*cis*-diamminedichloroplatinum(II)] (see Figure 1) has a significant activity in ovarian, testicular, bladder, head and neck, and lung cancer, where it is most commonly used in combination with other drugs [1]. Thus, it has become one of the most successful anticancer drugs used worldwide in almost 50% of solid tumor chemotherapies. Although the initial response rates can be high with cisplatin-based regimen, the clinical utility of the drug is often limited by the onset of acquired or intrinsic resistance [2, 3] and the number of side effects such as kidney damage, vomiting/nausea, and neurotoxicity [4]. The resistance of tumor cells to cisplatin remains a major cause of treatment failure in cancer patients, while the high toxicity of cisplatin limits the dose that can be given to patients. Cancer cell resistance to platinum chemotherapeutic drugs therefore remains a major obstacle and demonstrates a need for alternatives. For this reason, vast efforts are committed to develop novel platinum-based complexes which might overcome the shortcomings of cisplatin. The ultimate aim is to find and characterize platinum-based derivatives with higher antitumor activity, which display a more tolerable toxicologic profile and overcome resistance in many tumor types. A second alternative approach sought is to develop a more efficient combination treatment of biologic response modifiers together with chemotherapeutic platinum drugs.

The enormous efforts of a number of research teams have borne fruit in two derivatives, carboplatin (*cis*-diammine-(1,1-cyclobutanedicarboxylato)platinum(II)) and, more recently, the so-called “third generation” platinum drug oxaliplatin (*trans*—R,R.cyclohexane-1,2-diammine)oxalatoplatinum(II)). Both compounds are effective in colon cancer treatment and are currently clinically approved worldwide [5, 6]. Several other platinum-based drugs have gained regionally limited approval: Nedaplatin (Japan), SKI2053R (South Korea), and Lobaplatin (China).

Although over 1000 complexes have been studied so far, only 10–20% of them turned out to be active against cancer cells in preclinical studies [7]. These include, for example, satraplatin which is currently in the third phase of clinical evaluation [8]. A complementary approach also promises to employ biologic modifiers such as cytokines, lipids, and other molecules in order to increase the efficacy of both traditional and novel platinum-based drugs. Adjuvant biochemotherapy with cytokines, such as interleukin-2 or interferon-alpha may increase response rates in patients [9], thus improving their efficiency, although regimen treatments must be selected carefully in order to reduce undesirable effects. Similar to cytokines, lipids, and their derivatives, drugs interfering with metabolism of lipid compounds or dietary supplements may provide additional benefits during the treatment with platinum-based drugs, including reduced side effects of chemotherapy or increased toxicity towards tumor cells [10–14]. The present review is intended to provide an overview of some opportunities opened for innovative therapeutic strategies involving both traditional and novel platinum complexes, with particular emphasis on the novel class of anticancer platinum(IV) complexes with adamantylamine, which have been intensively studied in our laboratories during recent years.

## 2. DEVELOPMENT OF PLATINUM(IV) COMPLEXES WITH ADAMANTYLAMINE AND THEIR IN VITRO/IN VIVO EFFECTS

Recently, *Ž*ák et al. designed and synthesized a series of novel platinum(II) and platinum(IV) complexes with amino derivatives of adamantane as one of its ligands: (SP-4-3)-(1-adamantylamine)amminedichloroplatinum(II), coded as LA-9, and (OC-6-43)-bis(acetato)(1-adamantylamine)amminedichloroplatinum(IV), coded as LA-12 (see Figure 1 ). LA-9 represents a four-coordinate(II), and LA-12 an octahedral six-coordinate platinum complex containing a bulky nonleaving hydrophobic (lipophilic) ligand—adamantylamine [15]. Since the early studies by Rosenberg et al. [16] it has been known that platinum(IV) as well as platinum(II) complexes can display antitumor properties. In addition, platinum(IV) complexes are much more inert to ligand substitution reactions than are their platinum(II) counterparts [17], and it is generally accepted that reduction to the lower oxidation state platinum(II) is essential for the anticancer activity of the platinum(IV) complex [18, 19]. Currently, clinical trials of the platinum(IV) derivative LA-12 are in progress and some promising results have been shown. LA-12 has been evaluated in a panel of preclinical studies including *in vitro* and *in vivo* antitumor efficacy and toxicologic and pharmacokinetic studies [20, 21]. In both murine ADJ/PC6 plasmacytoma and a human A2780 ovarian carcinoma tumor model, higher *in vivo* antitumor activity of a single dose as well as of repeated doses was observed, compared to cisplatin and the other platinum(IV) complex—satraplatin (JM216, the first orally administered platinum(IV) drug currently evaluated in clinical trials) [21]. Interestingly, the acute toxicity of LA-12 in mice is relatively low and the effective dose is comparable to that of cisplatin and higher than that of satraplatin [21].

These highly interesting compounds were also successfully tested in our laboratories. We have demonstrated that LA-9 and LA-12 display a higher hydrophobicity than cisplatin and exert a higher cytotoxicity than satraplatin (JM216) in the *in vitro* tests on a panel of 14 cancer cell lines of various origin, and a different sensitivity to cisplatin, including both A2780 (cisplatin-sensitive) and A2780cis (with acquired cisplatin resistance) ovarian cancer cells, breast carcinoma, colon cancer, lung carcinoma, and leukemia cells lines, many of which are refractory to cisplatin treatment [22, 23]. In A2780cis-resistant cells, for example, the IC50 values were 1.4 and 24.23 *μ*M for LA-12 and cisplatin, respectively, with LA-12 being effective at the dose approximately equal to cisplatin IC50 in parental A2780 cells, thus proving that LA-12 may successfully counter the acquired resistence *in vitro* [22]. The IC50 value for LA-12 in A2780 was also approximatelly three times lower than IC50 of LA-12 in nontransformed rat liver epithelial cells, suggesting that it is not overtly toxic in nontumorigenic cells [24]. Interestingly, we found strong differences between the effects of platinum(IV) complex—LA-12 and platinum(II) derivatives—LA-9 and cisplatin on cytokinetic parameters in SK-OV-3 cells (with intrinsic cisplatin resistance) [25]. Apparently, both LA-12 and LA-9 are effective in considerably lower concentrations than cisplatin and can overcome both acquired and intrinsic resistance to cisplatin. Thus, the mechanisms of the effects of these compounds should be further investigated, as several recent studies have demonstrated that LA-12 may have unique effects which could be responsible for its high efficiency [24, 26].

## 3. A SEARCH FOR MECHANISMS OF ACTION OF NOVEL PLATINUM COMPLEXES

The activity of the “classical” cisplatin is generally accepted as being due to its ability to form adducts with DNA (inter- and intrastrand DNA cross-links) and causing DNA strand breaks in the nucleus [27, 28]. Subsequently, interference with normal transcription and/or DNA replication mechanisms [29] are its characteristic biological effects, leading to either repair of the DNA damage and cell survival, or activation of the irreversible cell death program as a consequence. Indeed, early observations have shown that cells deficient in DNA repair pathways are hypersensitive to cisplatin [29, 30], suggesting that cisplatin and other platinum-based complexes bind DNA.

On the other hand, cisplatin may react with many other cellular components that have nucleophilic sites such as RNA, proteins, membrane phospholipids, cytoskeletal filaments, and thiol-containing molecules [31]. Although cisplatin can react with a variety of cellular macromolecules and there is strong evidence that the most important target is DNA [27], it is estimated that less than 1% of the cisplatin molecules that enter the cell actually bind nuclear DNA, with most of them ending up binding other biomolecules [32]. This indicates that increased attention should also be focused on the discovery of mechanisms of action of platinum-based drugs, which are different from those primarily based on DNA damage. Such mechanisms could substantially interfere with the other intracellular signaling pathways regulating cell growth and death.

## 4. UNIQUE PROPERTIES OF NOVEL PLATINUM DERIVATIVES

The cytotoxicity of platinum-based anticancer drugs is therefore a result of many events, beginning with the intracellular accumulation and proceeding through detoxification by intracellular thiol-containing molecules, DNA modification, and cellular responses to DNA damage. Modification of the leaving group(s) in platinum complexes influences both biodistribution and the general toxicity. On the contrary, modification of the amine ligand(s) will influence the anticancer properties, since the nonleaving amine ligands are the reason for structurally different DNA or DNA/protein adducts. The hydrophobicity (lipophilicity) and bulkiness of the nonleaving amine ligand significantly affect several factors (permeation through the cell membrane, reactivity towards DNA and thiols, and recognition of DNA adducts) that are responsible for cytotoxic potential of platinum complexes on the molecular and cellular levels. In various platinum(II) complexes, the increase of ligand hydrophobicity is positively correlated with the increase of the cytotoxic effect [33].

It is therefore interesting that a similar correlation was found within a homologous series of platinum(IV) complexes [23]. The higher hydrophobicity of the platinum complexes enhanced their penetration through the cell membrane and increased their accumulation inside the cancer cells. The high intracellular concentrations of platinum(IV) drugs, together with their reactivity towards DNA, may accelerate the production of various platinum-DNA or platinum-DNA/protein adducts, which are more or less endowed with the ability to trigger apoptosis or necrosis in target cells. The hydrophobic complex LA-12 is a good example of a platinum(IV) complex with a bulky hydrophobic ligand, enhancing accumulation in cancer cells and triggering rapid cell death in both cisplatin-sensitive and cisplatin-resistant cell lines [22, 25]. LA-12 proved to be very toxic against various cancer cells representing different types of cancer and employing different mechanisms of resistance towards cisplatin [23].

In comparison to cisplatin and satraplatin (JM-216), LA-12 represents a complex with a very high hydrophobicity due to the presence of adamantylamine ligand. Moreover, the symmetry of the hydrophobic adamantyl moiety lightens penetration of the whole complex through biological membranes. This theoretical presumption has been proven experimentally in cisplatin-sensitive and cisplatin-resistant cell lines A2780 and A2780/cisR, respectively. Platinum uptake in sensitive and resistant A2780 cell lines was more than one order of a magnitude lower for cisplatin in comparison with LA-12. Moreover, accumulation of LA-12 in cisplatin-resistant cells was not decreased in the range having an effect on biological activity [26].

### 4.1. Effects of LA-12 in cisplatin-resistant cells

In all human cancer cell lines studied so far, it seems apparent that cisplatin resistance is a multifactorial process, being a result of decreased drug uptake, increased DNA repair and tolerance to DNA lesions, slow cell proliferation and replicative bypass, and/or cytosolic drug inactivation. We cannot exclude that LA-12 is a substrate for some of multidrug resistance proteins (MRP; MDR) and P-glycoprotein (P-gp), but the low cross-resistance with cisplatin together with high accumulation in cisplatin-resistant cancer cell line A2780/cisR seem to imply that MRP/MDR systems are of secondary importance in comparison with increased ability of hydrophobic LA-12 to penetrate through biological membranes.

Ovarian epithelial cancer cell line A2780 is a well-described model for studies on cisplatin resistance. The resistant sublines, prepared by gradual treatment by cisplatin, are available and several mechanisms of resistance have been described. It seems that the cisplatin-resistant A2780 sublines represent the type of cisplatin-resistant cells, in which several mechanisms of resistance are well orchestrated. An organic anion pump that could be involved in cisplatin resistance is a multidrug resistance protein 2 (MRP2). High expression of MRP2 was found in A2780/cis70 cell line, together with increased levels of glutathione [34]. Cisplatin-resistant A2780/cis70 cells demonstrated enhanced efflux of ciplatin as compared to parent A2780 cells. Enhanced DNA repair was also observed in A2780/cis70 cells and this mechanism has been shown to be responsible for cisplatin resistance [35]. Expression of all the above-mentioned systems gives the cisplatin-resistant A2780 sublines a remarkable capability to survive the attack of cisplatin. LA-12 demonstrated also very low cross-resistance in both A2780/cis40 and A2780/cis90 sublines. There was no cisplatin cross-resistance with intermediate resistant A2780/cis40. In contrast, cross-resistance has been observed for another new generation platinum(II) complex ZD0473 (formerly known as JM473, AMD473) in A2780 and A2780/cis40 cells [36]. Interestingly, we have observed a substantial cross-resistance for satraplatin (JM-216), using a panel of cisplatin-resistant cancer cell lines (see Table 1).

Strong inhibition of DNA polymerization by LA-12/DNA adducts, enhanced persistence of these adducts due to their less efficient repair, and DNA-protein cross-linking could be responsible for the unique cytotoxic activity of LA-12 [23, 26]. Therefore, increased attention should be paid to this compound, with the aim to define its mechanisms of action, as well as to search for alternative approaches improving its anticancer activity. In the following sections, we summarize the current possibilities of modulating the effects of cisplatin, which might also be employed for novel platinum-based drugs, such as platinum(IV) derivatives with adamantylamine. Our attention is primarily focused on signaling pathways regulating the programmed cell death (apoptosis) and modulation of cellular lipids. For more information on other drug-resistance mechanisms, such as MDR, the readers are kindly requested to consult several recent excellent reviews [37–39].

## 5. ACTIVATION OF SIGNALING PATHWAYS LEADING TO CELL DEATH

In general, apoptosis is the most important form of cell death induced in response of tumor cells to chemotherapeutic agents, including platinum-based cytostatics. However, in some cell lines, particularly those with resistance to the drug, platinum-derived chemotherapeutics may also produce characteristic features of necrotic cell death. Moreover, in the same population of cisplatin-treated cells, necrotic and apoptotic cell deaths have been shown to occur simultaneously (for review see [40]). In addition, other death signaling pathways may also be stimulated following cisplatin treatment, finally leading to autophagy [41]. Thus, different types of cell death induced by the chemotherapeutic drugs have been reported to occur, depending on the cell type and/or drug concentration.

### 5.1. Apoptotic pathways regulated by anticancer drugs

Two major distinct apoptotic pathways have been described for mammalian cells. The intrinsic pathway is characterized by the central role of the mitochondria in the apoptotic signaling cascade. Bcl-2 family proteins are known as important regulators at the level of these organelles. Translocation of proapoptotic proteins from mitochondria to the cytosol results in caspase activation and execution of apoptosis. This pathway functions in response to various types of intracellular stress-inducing agents, DNA damage, growth factor withdrawal, or in response to activation of death receptor-mediated apoptosis in type II cells. On the other hand, in the extrinsic pathway, caspase activation is initiated by the death receptors on the surface of the so-called type I cells and this pathway may proceed independently of mitochondria [42]. Chemotherapeutic agents are known to induce/modulate apoptosis at the level of both intrinsic and/or extrinsic pathway.

Emerging evidence suggests that defects or dysregulation of different steps of the apoptotic signaling pathways may be an important determinant of resistance to anticancer drugs (reviewed in [43, 44]). The mechanisms that inhibit propagation of the apoptotic machinery induced by cisplatin may include loss of p53 function, overexpression of Her-2/neu, activation of PI3K/Akt pathway, overexpression of antiapoptotic Bcl-2 protein, interference in caspase activation, and so forth. The molecular signature defining the resistant phenotype varies among tumors, and the number of resistance mechanisms activated in response to selection pressures dictates the overall extent of cisplatin resistance (for review see [28]).

### 5.2. Interactions of platinum-based drugs and death ligands

The onset of resistance or cross-resistance of tumor cells to several chemotherapeutic drugs is a serious therapeutic complication. Different agents have been investigated with regard to their ability to induce/modulate signaling pathways involved in drug-induced apoptosis. Among them, members of the tumor necrosis factor (TNF) family of apoptotic inductors are of particular interest. These agents have been shown to be capable of triggering/modulating mitochondria-dependent and/or mitochondria-independent apoptotic pathways inside the cell and may contribute to the potentiation of apoptosis, when coadministered together with chemotherapy [45]. Elucidation of the mechanisms involved in these effects therefore deserves further attention.

A number of studies, including our own preliminary data, have shown that TNF-related apoptosis-inducing ligand (TRAIL) (an important member of the TNF family) works synergistically with some anticancer drugs to kill many types of tumor cells and eliminate resistance to such drugs. These findings indicate that the combination of TRAIL and chemotherapeutic drugs is potentially promising in treating refractory cancers. TRAIL can induce human cancer cell apoptosis through the engagement of its death receptors (DRs) DR4 and DR5. However, not all cancers are sensitive to this cytokine. The mechanisms of modulation of the death receptor-mediated apoptosis (induced by TRAIL) and DR-mediated regulation in colon cancer cells have also been studied in our laboratory [46, 47]. Recently, it has been shown that low doses of cytotoxic drugs could restore TRAIL-induced cell death in resistant colon and prostate cancer cell lines [1, 48]. Ligation of TRAIL to its cognate receptors results in receptor activation and formation of a death-inducing signaling complex (DISC), which is responsible for caspase-8 activation and further propagation of the apoptotic signal [42]. Chemotherapeutic agents have been shown to have a great impact on these processes.

At the level of plasma membrane, upregulation of the DRs was shown to be responsible for cisplatin-induced sensitization to DR-mediated apoptosis [49]. Chemotherapeutic agents can induce a redistribution of CD95, DR4, and DR5 in lipid rafts that accounts for the synergistic toxicity of chemotherapy and DR ligands in colon carcinoma cells [50]. An increased recruitment of Fas-associated death domain (FADD) adaptor protein and procaspase-8 to the TRAIL DISC was demonstrated in colon cancer cells exposed to cisplatin-based drugs [51]. Interestingly, phosphorylated FADD was shown to play an essential role in the mechanisms of amplification of chemotherapy-induced apoptosis in prostate cancer cells [52]. Furthermore, cisplatin downregulated the levels of c-FLIP_L_ (a competitive inhibitor of caspase-8 at the DISC level) in osteosarcoma cells, sensitizing them to Fas-mediated apoptosis [53].

The cisplatin-mediated potentiation of TRAIL-induced apoptosis has been replaced at the mitochondrial level, as demonstrated by Bid cleavage, the dissipation of mitochondrial membrane potential (MMP), and the release of mitochondrial proteins into the cytosol of the mesotheliomal cells [54]. Exposure of esophageal cancer cells to sublethal concentrations of cisplatin resulted in profound potentiation of their susceptibility to TRAIL cytotoxicity, accompanied by significant activation of caspase-8, -9, and -3. Interestingly, activation of these caspases was abrogated by overexpression of Bcl-2 or by the selective caspase-9 inhibitor, which pointed to the essential role of mitochondria-dependent signaling cascade in this process [55]. Similarly, the mitochondria-dependent caspase activation cascade has been shown to play a significant role in cisplatin-mediated potentiation of TRAIL-induced apoptosis in thoracic cancer cells [56]. These results suggest that mitochondria could be a direct target for the development of more refined strategies to enhance the therapeutic effect of TRAIL as an anticancer agent.

## 6. CHEMOTHERAPY AND MITOCHONDRIA

Comparison between cisplatin, oxaliplatin, and carboplatin effects on cancer cells shows that, in addition to the extent of nuclear DNA platination and fragmentation, impairment of mitochondrial functions could also be a measure of platinum drug cytotoxicity [57]. Cisplatin has been shown to accumulate in mitochondria of cultured cells and to have effects on isolated mitochondria depending on cell type. Cisplatin and its second generation derivatives produced marked changes of mouse live mitochondrial morphology, enzyme activity, Ca^2+^ influx, and surface potential. However, these effects did not correlate with nephrotoxicity [58]. Cisplatin binds to kidney mitochondrial proteins in treated mice and inactivates the alpha-ketoglutarate dehydrogenase complex in cultured LLC-PK1 cells, which is associated with drug bioactivation [59]. Evaluation of mitochondrial oxygen consumption using various cell lines and tumors from patients showed that cisplatin does not directly damage the energy-converting mechanism of mitochondria [60]. In 3T3 and Walker 256 cells, cisplatin had acute, but reversible, effects on the reorganization of the intermediate filament component of cytoskeleton as well as mitochondrial function [61]. Using head and neck squamous cell carcinoma cell lines, it has been shown that cisplatin forms adducts with mtDNA more abundantly than those with nuclear DNA and binds preferentially to mitochondrial membrane proteins [62]. Changes of mitochondria and their function are associated mainly with induction of intrinsic apoptotic pathway. Alteration on mitochondria membrane level (dissipation of MMP), release of mitochondrial proteins into cytosol, and production of reactive oxygen species are associated with activities of Bcl-2 family proapoptotic and antiapoptotic proteins and activation of caspases [62].

In various types of cancer cells, mitochondrial alterations have been shown to be associated with platinum-drug resistance. The mitochondria of intrinsically resistant human ovarian cancer cell lines and resistant clones of HeLa cells showed altered morphology and were fewer in number. In these models, cisplatin resistance was associated with lower platinum accumulation and mitochondrial defects [63]. It has been shown that density of mitochondria in intestinal epithelial cells is the key factor for the determination of the anticancer activity and side effects of cisplatin [64]. Next, elevation of MMP in ovarian cancer cells with acquired platinum resistance has been observed, suggesting that changes at the mitochondrial level would affect the relative resistance of malignant cells to undergo drug-induced apoptosis [65]. Impairment of mitochondrial apoptotic signaling pathway is responsible for incomplete caspase-3 activation and lower sensitivity of gastric cancer cells to cisplatin [66].

The upregulation of the antiapoptotic Bcl-2 protein contributed to the development of cisplatin resistance, which was reversed by Bcl-2 siRNA [67]. On the other hand, targeted inactivation of proapoptotic Bax in colon cancer HCT116 cells resulted in a significantly increased resistance to cisplatin [68]. Failure of caspase-9 activation induced a higher threshold for apoptosis and cisplatin resistance in testicular cancer [69]. Resistance to apoptosis correlated with the reduced caspase-3 activation and enhanced the expression of antiapoptotic proteins in human cervical multidrug-resistant cells [70]. Furthermore, caspase-2 is known to mediate the recruitment of the mitochondrial apoptotic pathway upon DNA damage induced by chemotherapy (reviewed in [44]). Cisplatin has been shown to induce p53-mediated caspase-2 activation resulting in the mitochondrial release of the apoptosis-inducing factor (AIF) and subsequent apoptosis in renal tubular epithelial cells [71]. From the information described above, it follows that the knowledge of platinum drug effects on mitochondria may help to design strategies for increase of the effectiveness of tumor cell treatment. Importantly, similar approaches might be potentially used to improve efficacy of both currenly used platinum derivatives and novel compounds, such as LA-12.

## 7. CHEMOTHERAPY AND OXIDATIVE METABOLISM

Studies in a variety of cell types have suggested that chemotherapeutic drugs induce cancer cell apoptosis at least in part by inducing formation of ROS. In several cancer cell models it has been demonstrated that cisplatin promotes increased production of ROS, which can activate nuclear factor-kappaB (NF-*κ*B). This can lead to increased expression of proinflammatory mediators and intensify the cytotoxic effects of cisplatin [72]. A positive correlation has been reported between lipid peroxidation and the declined survival rate of murine melanoma cells after treatment with novel platinum(II) complexes [73]. On the other hand, in B lymphoma cells the cisplatin-induced apoptosis occurs via a mechanism not involving oxidants [74]. Similarly, in mouse fibrosarcoma and human astrocytoma cells, cisplatin caused oxidative stress-independent apoptotic cell death. Importantly, in the same cell systems, overproduction of ROS was detected after treatment with the novel platinum(IV) complex with a higher cytotoxicity compared to cisplatin, which induced primarily necrosis [75].

Nevertheless, the role of oxidative metabolism associated with the action of metal-based cytostatics is still rather controversial. Production of ROS and generation of lipid peroxides (LP), and thus induction of oxidative stress, are considered to be some of the main causes of metal-based drug organ toxicity, especially nephrotoxicity and hepatotoxicity [76]. Antioxidants can reduce or prevent many of these side effects. In this context, the beneficial effects of compounds with antioxidative properties like resveratrol or green tea have been demonstrated [77].

It seems evident that the production and role of ROS and LP in the effects of platinum drugs may depend on the structure and concentration of the drug, the cellular system used, the capacity of the antioxidant system, and can be further modulated by the presence of other factors like TNF, which may alter oxidative/antioxidative balance [78].

## 8. INTERACTIONS OF PLATINUM-BASED DRUGS WITH CELL MEMBRANES AND WITH LIPID SIGNALING/METABOLISM

### 8.1. Impact of polyunsaturated fatty acids (PUFAs) on anticancer drug effects

There is increasing interest in the new therapeutic approaches based on the knowledge of structure, function, and alteration of membrane lipids [79]. Experimental data *in vitro* and *in vivo* and also several preclinical studies have shown that certain PUFAs, especially of omega-3 type (found in marine plankton, fish oil, and some plant oils), possess anticarcinogenic activities including suppression of neoplastic transformation, cell growth inhibition, enhanced apoptosis, and antiangiogenicity especially in colon, breast, and prostate tissues. This topic is behind the scope of our review and many reviews have been published until now [80–82]. Therefore, we pay attention mostly to the mechanisms, which could be considered in possible anticancer strategy using combined treatment with cytostatics and PUFAs.

Several molecular mechanisms whereby omega-3 PUFAs may modify carcinogenic process have been proposed. These include suppression of biosynthesis of arachidonic acid-derived eicosanoids (especially due to decreased COX-2 expression and activity), modulation of membrane properties and signal transduction pathways, effects on transcription factor activity, gene expression, alteration of estrogen metabolism and insulin sensitivity, and increased or decreased production of reactive oxygen and nitrogen radicals [83, 84] (for more details see below). Supplementation of tumors with long-chained omega-3 PUFAs results in enrichment of tumor phospholipid fractions with these PUFAs. Such cells then possess membranes with increased fluidity, an elevated unsaturation index, enhanced transport capabilities that result in accumulation of selective anticancer agents, increased activity of selected drug-activating enzymes, and alteration of signaling pathways important for cancer progression. These changes may enhance tumor responsiveness to antineoplastic agents such as doxorubicin, mitomycin C, or 5-fluorouracil [10, 85, 86], especially in tumor lines that are resistant to chemotherapy. Studies of associations between the levels of fatty acids, especially docosahexaenoic acid (DHA) stored in breast adipose tissue and the tumor response to various chemotherapeutic agents in breast carcinoma patients suggest that increased DHA content may improve therapeutic effects [87]. There are several reports demonstrating possibilities of enhancement of cisplatin cytotoxicity and modulation of cisplatin resistance by PUFAs in ovarian A2780, breast MCF-7, and small cell lung GLC4-sensitive and GLC4-resistant cancer cell lines [11, 12, 88]. The anticancer activity of cisplatin has been shown to be effectively supported by fish-oil diet under a conditioned balance of oxidation and antioxidation adjusted by vitamins E and C [89]. Therefore, it appears that dietary administration of omega-3 PUFAs might be a relatively nontoxic form of cancer-supporting therapy increasing the effects of various anticancer agents including those based on platinum. In addition, certain PUFAs may also prevent or reduce some of the side effects of these therapies and tumor cachexia [90]. Therefore, it is important to study the mechanisms of the effects of PUFAs and other lipid molecules and to look for their links with cytostatic drug effects.

### 8.2. Cell membrane properties and drug effects

It has been shown that the levels of lipids in cell membranes of cancer patients and in cancer cells resistant to chemotherapy are altered [91, 92]. Nevertheless, the importance of cell membrane composition/properties and their modulation for the effects of platinum drugs is not clear. It has been found that cisplatin-resistant epidermal cells have a higher membrane potential and more fluid membranes than cisplatin-sensitive cells [93]. On the other hand, no differences have been found in major phospholipids (PLs) or in free cholesterol content between cisplatin-resistant and cisplatin-sensitive human ovarian carcinoma cells. The decreased cisplatin accumulation in resistant cells is probably not due to membrane changes, which might lead to retardation of passive diffusion of the drug into the cells [94]. It has been demonstrated that cisplatin may interact and form complexes with negatively charged PLs like phosphatidylserine and could change fluidity of the membranes [95, 96]. Modulation of membrane lipid composition and metabolism may be important for cell behaviour, especially in tumor cells, as their cell membranes are characterized by changes in PL constituents and membrane fluidity, namely, by a decreased content of PUFAs [97]. All above studies seem to suggest that modulation of cell membrane composition and functions might modulate the effects of platinum-based drugs.

### 8.3. Mechanisms of PUFAs effects

The mammalian organism is not able to synthesize PUFAs and thus they have to be supplied in the diet. These essential PUFAs play an important role in transducing signals from the extracellular space and function as inter- and intracellular mediators and modulators of the cellular signaling network [98]. Their effects on various levels of cell organization and their interaction with other endogenous or exogenous factors can finally significantly influence cell proliferation, differentiation, and apoptosis [99].

The mechanisms of action of PUFAs are not yet fully understood and are likely to be very complex. The main mechanisms at the cellular level might include:

1. incorporation of PUFAs into cancer cell membranes, thus altering their physical and functional properties and modulating signaling pathways involved in the regulation of cytokinetics; changes in membrane composition further determine the ability of associated proteins, such as receptors, ion channels, and so forth, to move and interact;
2. alteration of cell oxidative metabolism due to lipid peroxidation and production of ROS or reactive nitrogen species (RNS);
3. conversion to their reactive and biologically active metabolites (e.g., eicosanoids);
4. activation of specific nuclear receptors and transcription factors leading to altered expression of genes involved in proliferation, differentiation, and apoptosis (cytokinetics);
5. interaction with other signal transduction pathways.

The most important of the above mechanisms are discussed below.

### 8.4. PUFAs and lipid effects on cell membrane level

As components of membrane PLs, PUFAs cause changes of membrane fluidity and remodeling of membrane structure, which may influence receptor-ligand interactions, signal transduction properties, and various aspects of membrane-mediated cellular functions [100].

PUFAs, particularly DHA as the longest and most unsaturated acyl chain commonly found in membranes, are thought to be essential components of lipid rafts which represent small dynamic plasma membrane microdomains of tightly packed proteins and lipids enriched with cholesterol and sphingolipids [101, 102]. These structures are functionally implicated in the compartmentalization, modulation, and integration of cell signaling, thus modulating important processes including cell growth, survival, and adhesion [103]. Sphingolipids constitute a class of lipids that may function as second messengers in different cellular processes as cell differentiation, growth, and death. In particular, ceramides have been implied in intracellular signal transduction systems regulating cellular differentiation, survival, and apoptosis, and thus appear capable of changing the lifestyle of virtually any cell type. Their production by sphingomyelinases (SMase) can play a pivotal signaling role through direct interaction with signaling proteins or through facilitating the formation and trafficking of signal transduction complexes [104]. As components of lipid rafts, EPA and DHA might increase the activity of SMase and the production of ceramide in breast cancer cells simultaneously decreasing the level of EGF receptors in lipid rafts, thus altering EGFR signaling and inhibiting cancer cell growth [105]. Apoptotic stimuli, such as death ligands or chemotherapeutic agents, can also activate SMases and generate ceramide [106]. Based on these findings, PUFAs might modulate function of proteins bound to membrane lipid domains including the transporter function of proteins like P-glycoprotein (P-gp). The lipid phase of the plasma membrane plays an important role with respect to MDR and P-gp, whose activity is highly sensitive to lipid environment. P-gp may also be involved in lipid trafficking and metabolism. PUFAs play a specific structural function in membranes and are capable of positioning a double bond into the inner leaflet of a membrane. There is not much information about direct effects of PUFAs on P-glycoprotein expression and activity. The available data suggest that DHA enhanced bioavailability of CYP3A substrates in the rat intestine, but did not inhibit P-gp [107]. In mouse leukemia cell line P388 GLA and DHA pretreatment improved doxorubicin (DOX) cytotoxicity due to enhanced DOX accumulation and changes of antioxidant enzyme activities. However, no changes of P-gp expression were observed [108]. Promising results were achieved using fatty acid diesters, which are more potent in reversing MDR *in vitro* due to their direct P-gp binding properties [109].

### 8.5. PUFAs and mitochondria

Apart from the cytoplasmic membrane, PUFAs also physically interact with mitochondrial membranes and alter their permeability by opening the permeability transition pores and decreasing MMP [110]. Apoptotic signals depend on redox catalytic interactions of cytochrome c with main mitochondrial PLs: cardiolipin and phosphatidylserine [111]. It has been demonstrated that in colonocytes, DHA is preferentially incorporated into cardiolipin, which coincides with increasing unsaturation, induction of oxidative stress, release of cytochrome c, and apoptosis [112]. PUFAs have also been shown to significantly modulate the level of several Bcl-2 family proteins (e.g., Bid, Bcl-2) interacting with mitochondrial membrane lipids which regulate apoptosis; cardiolipin has been proposed to be a “lipid receptor” of Bid [113].

### 8.6. PUFAs and oxidative metabolism

Antiproliferative and apoptotic effects of PUFAs were shown to be associated with changes on mitochondria and induction of oxidative stress. Susceptibility of their double bonds to oxidation is the reason for production of various types of biologically active metabolites like eicosanoids, ROS and RNS, and lipid peroxides (LPs) [114]. We together with many others have shown that supplementation of cultured cells with PUFAs dose-dependently enhanced ROS production and lipid peroxidation [115, 116]. Many studies have documented that PUFAs augmented free radical generation and formation of LPs selectively in the tumor cells compared to normal cells despite the fact that the uptake of fatty acids in the tumor cells was lower [117]. There is a close correlation between the rate of lipid peroxidation and the degree of malignancy of tumor cells, and the susceptibility to ROS-induced cytotoxicity. Dividing cells show low levels of lipid peroxidation as compared to slowly dividing or nondividing cells. Resistance to lipid peroxidation appears to occur at the premalignant stage of the carcinogenic process. ROS also serve as common messengers downstream of various stimulus-specific pathways leading to NF-*κ*B activation, including TNF-*α*, PUFAs, or cyclooxygenases (COXs), and are involved in the regulation of immune response, proliferation, and apoptosis [118, 119].

Taken together, ROS and LPs can function as a “double-edged sword.” Their excess may damage normal cell populations, while they may have antiproliferative and apoptotic effects in cancer cell population [97]. These events may interfere with the modulation of oxidative metabolism induced by platinum-based anticancer drugs, as described earlier.

### 8.7. Effects of PUFAs on signal transduction and gene expression

PUFAs and their metabolites are involved in many intracellular signaling pathways activating or inhibiting various components of this machinery and modulate gene expression [120]. They may alter the levels and signaling pathways of various mediators, such as eicosanoids and cytokines [46, 115, 121].

The increase of phosphatidylserine by DHA resulted in a modification of the PI3K/Akt pathway, which is a major signaling pathway mediating proliferative and differentiation signals in many cell types *in vitro* and *in vivo*. *ω*-3 PUFAs decrease cell proliferation and induce apoptosis possibly by decreasing signal transduction through the Akt/NF-*κ*B cell survival pathway. In addition, PUFA-mediated modulation of other signaling pathways, such as those involving protein kinase C, extracellular signal-regulated kinase, and p38 mitogen-activated protein kinase, has been reported [122]. Fatty acids and their metabolites are generally considered as agonists of peroxisome proliferator-activated receptors (PPARs). Furthermore, PUFAs can also interact with other transcription mediators including NF-*κ*B [120]. PPAR and NF-*κ*B activation can be further associated with the regulation of expression of COX-2, an enzyme playing an important role in many pathological conditions [123].

Several studies using *in vitro* cell cultures have confirmed that certain PUFAs have a selective cytotoxic or antiproliferative effect on tumor cells and a minimal or no effect on normal cells. However, often cells from different species and different tissues were compared. The effects of PUFAs also depend on time of treatment, concentration, and culture conditions such as growth rate, cell confluency, type of medium, and amount of serum [124]. Nevertheless, it has been shown that the fatty acid composition of tumor tissues differs from normal ones and that also activities of fatty acid metabolism enzymes and oxidative defence were distinct. Tumor cells are characterized by the loss or decreased activity of desaturases and thus a decreased metabolism of precursor omega-6 linoleic acid and omega-3 *α*-linolenic acid to longer chain fatty acids such as AA or EPA and DHA. They also have a low level of cytochrome P450 enzymes which can initiate and propagate lipid peroxides. Moreover, malignant cells have markedly enhanced levels of antioxidants and low levels of superoxid dismutase, glutathione peroxidase, and catalase enzymes. These properties may be prerequisite for tumor-selective augmentation of free radical generation and formation of lipid peroxides compared to normal cells after exogenous addition of PUFAs. These products of PUFA oxidative metabolism are powerful inducers of p53 activity, influence the expression and activity of members of the Bcl-2 family, activate caspases, and shorten telomeres, thus inducing apoptosis of cancer cells. They were also shown to play a role in the metastatic process and angiogenesis [97]. Recently developed animal models can closely mimic the clinical course of carcinogenesis and showed that it is especially the ratio of omega-3 to omega-6 PUFAs which plays an important role in cancer development [125]. The data from multiorgan carcinogenesis model in male rats treated with various types of PUFAs provide evidence of their organotropic effects on carcinogenesis, which correlates with reduction of tissue AA levels in the target organs [126]. It was reported that PUFAs are potent inhibitors of hepatic glycolysis and de novo lipogenesis and promote a shift from fatty acid synthesis and storage to oxidation inhibiting lipogenic genes through specific transcription factors SREBP-1 and ChREBP [127]. Thus, further studies evaluating especially the effects of PUFAs on tumor versus normal cells and tissues using *in vivo* systems are necessary before copper-bottomed therapeutic use of omega-3 PUFAs for cancer patients. The future promising approaches include use of new PUFA analogues [109] and the development of specific conjugates of PUFAs with cytostatic drugs. In tumor-bearing mice, it has been demonstrated that conjugates of DHA with taxanes are stable in plasma for a long time, are less toxic than taxanes alone, and posses significantly increased antitumor activity [128, 129]. Recently, the new conjugates of second generation of taxoids with DHA exhibited strong activity against drug-resistant colon cancer and drug-sensitive ovarian cancer xenografts in mice [130] .

## 9. TARGETING OF CISPLATIN-BASED DRUGS

New strategy of development of PUFA-anticancer drug conjugates might be based on the fact that some PUFAs are taken more rapidly by tumor cells than by normal cells. The new DHA-taxoid conjugates exhibit a strong activity against drug-resistant colon cancer and drug-sensitive ovarian cancer xenografts in mice and a reduced systemic toxicity as compared to taxoids alone [130]. It might be speculated that agents with higher lipophilicity, such as LA-12, would be more effective in suppression of proliferation and induction of cell death in the conditions of modulated phospholipid metabolism. The above-mentioned effects should be taken into account when developing new strategies targeting platinum-based drugs. Encapsulation in liposomes and linking of cytotoxic platinum drugs to macromolecular carriers are strategies for targeting to solid tumors. Effective accumulation of drug carriers in the tumor requires a long half-time in circulation as well as an appropriate size of carriers (liposomes or polymers), which should be below 120 nm to penetrate into the tumor tissue through fenestrations in the vasculature of capillary vessels nourishing the tumor. The extracellular matrix represents another hindrance in the penetration of drug carriers deep into the tumor tissue [131]. A liposomal cisplatin preparation SPI77 has been studied in both pediatric and adult patients, but the liposomes used were too stable and cisplatin was insufficiently released from them [132]. An improved liposomal cisplatin termed lipoplatin [133] is about to enter phase III studies, and liposomal oxaliplatin is expected to enter the clinical stage of development in the near future [134].

Several cisplatin derivatives with long alkyl chains were prepared to improve their direct incorporation into the liposomal membrane. Preliminary evidence of antitumor activity of L-NDDP (liposomal formulation of *cis*-bis(neodecanoato)(*trans*-R,r-cyclohexane-1,2-diamine)platinum(II)) was reported from a phase II trial in refractory metastatic colorectal cancer [135]. Nevertheless, clinical development has been halted in order to improve drug formulation.

## 10. OTHER MECHANISMS

Although this review is dedicated principally to the role of lipids and possibilities of modulating the effects of novel platinum derivatives, we would also like to briefly touch the issues of inhibition of intercellular communication and possibilities of modulation of multidrug resistance.

### 10.1. Modulation of gap-junctional intercellular communication by platinum derivatives

Intracellular communication through gap junction (GJIC) has been shown to be crucial for both tissue and cellular homeostasis. Disruption of GJIC through defects in expression, posttranslational modification, and localization of connexins and/or mutation of genes coding for connexins seems to be one of the mechanisms involved in carcinogenesis [136]. Many chemopreventive and antitumor agents upregulate GJIC, which can be a significant mechanism of their effects [137]. On the other hand, tumor promoters (e.g., phorbol esters, dithiothreitol) and also some anticancer drugs such as doxorubicin, taxol, and also cisplatin decrease GJIC [138–140]. Our own data indicate that disturbance of tissue homeostasis owing to inhibition of GJIC may be linked to a direct cytotoxic effect of platinum drugs, rather than an indirect effect found for some carcinogens (e.g., TPA) or polyaromatic tumor promoters [24]. It has been shown that the increased level of connexin 43 may significantly improve the efficiency of anticancer therapy [141–143]; the level of connexin 43 expression correlates with the sensitivity to anticancer therapy and the quality of regeneration after the therapy [141, 144]. These studies strongly support the importance of “bystander” cells and functional GJIC in cancer therapy and recovery [145, 146]. Interestingly, it has been shown that cisplatin can induce cell-interdependent cell death through Ku/DNA-dependent protein kinase signaling in mouse embryonic fibroblasts [147]. Inhibition of GJIC might thus significantly limit its efficiency in target cells, suggesting that restoration of GJIC in tumor cells or prevention of inhibitory effects of anticancer drugs on GJIC might increase the effectiveness of both established and novel platinum derivatives. This area of research presents a challenge for the development of new drugs and/or treatment protocols, which would lead to elimination of these side effects.

### 10.2. Role of resistance in response to platinum derivatives

Resistance is a major problem in the application of platinum-based therapy. The postulated mechanisms of cisplatin resistance include reduced drug accumulation by changing the profile of uptake/efflux, inactivation of cisplatin by increased levels of intracellular thiols such as glutathione, metallothionein, or other sulfur-containing molecules, increased repair of cisplatin adducts, increased tolerance to cisplatin adducts, and failure of apoptotic response. Reduced intracellular drug accumulation in resistant cells might be ascribed to an inhibition of drug uptake, an increase in drug efflux, or both. Resistance to cisplatin can be acquired through chronic drug exposure or it can present itself as an intrinsic phenomenon. The results from our laboratory showed a great potential of LA-12 to overcome both acquired [22] and intrinsic resistance in ovarian cancer cells [25]. The mechanisms enabling LA-12 to defeat platinum resistance are currently unknown. However, they might be related to a number of events, including enhanced accumulation of the drug, strong inhibition of DNA polymerization, decreased DNA repair, and formation of DNA-protein cross-links as compared to cisplatin [26].

The development of alternative platinum analogues or their application, in combination with drugs acting through different mechanisms, seems to be an effective strategy, which would help to solve problems of cisplatin resistance. However, this strategy usually fails in cases when the resistance of the cells is due to their inability to die by apoptosis, which is not directly drug-dependent [148]. Interestingly, LA-12 is able to induce not only apoptosis, but also a caspase-independent type of cell death [25]. This could be an advantage when using this novel platinum(IV) agent for the treatment of tumors with defects in the classical apoptotic pathway.

Another strategy to address the problem of cisplatin resistance is drug resistance reversal therapy [149]. This therapy is simply focused on restoration of sensitivity to platinum-based drugs through targeting of known mechanisms of resistance using specific inhibitors of influx, efflux channels and transporters, glutathione-S-transferase, DNA repair, p53 pathway, and so forth. It is currently not known whether such strategies would also increase the efficiency of LA-12 and related compounds.

## 11. CONCLUSION

**Figure 2:**
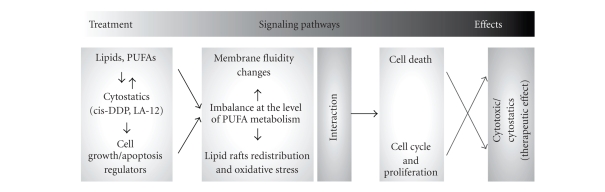
A diagram depicting potential innovative chemotherapy strategies which might be employed to increase efficacy of novel platinum(IV) complexes.

More than thirty years of experimental and clinical studies on platinum-derived compounds have led to the development of potent anticancer drugs. However, despite the progress within this field, treatment of generally more resistant types of cancer, such as colon or prostate cancer, by more efficient cisplatin compounds still remains elusive. Recently developed platinum(IV) complexes with adamantylamine have the potential for a significantly improved therapy of many types of tumors. Based on the findings discussed above, we propose that several innovative approaches might be used to modulate the anticancer effects of platinum(IV) complexes with adamantylamine. These may include, for example, combined application with PUFAs and/or death ligands, or modulation of intercellular communication. Although sufficient data on the role of endogenous regulators in the mechanism of action of platinum-based drugs in the regulation of mammalian cell kinetics (proliferation, differentiation, and apoptosis) are currently limited, the role of PUFAs in modulating these processes should be emphasized, as summarized in Figure 2. A possible potentiation of platinum-based drug effects by relatively cheap PUFAs from the diet and/or lipid preparations would allow to decrease the doses of expensive antineoplastic drugs, thus preventing side effects and allowing to use them also in low-sensitivity tumor types. Further studies are needed to address these questions and to understand the mechanisms involved.

## Figures and Tables

**Table 1:** Comparison of the cytotoxicity of cisplatin, LA-12 and satraplatin (JM-216) against cisplatin-resistant tumor cell lines. Values of IC_50_ were calculated for 24 hour exposure time (adapted from Turánek et al. Anticancer Drugs. 2004,15:537-543).

Cancer cell line	IC_50_ (*μ*M )		
	Cisplatin	LA-12	Satraplatin
K562	>80	3	63
KG-1	48	2	63
ML-2	>80	1	56
B16	>80	6	>80
HT-29N	>80	12	>80
HT-29	50	8	70
HCT116	>80	9	>80
A427	63	6	13
HBL100	63	6	>80
MCF-7	71	8	70
CPRL23/CTR	>80	25	56
A2780	7	4	63
A2780cis40	40	3	40
A2790cis90	>80	7	63
